# CD20 × CD3 bispecific antibodies for lymphoma therapy: latest updates from ASCO 2023 annual meeting

**DOI:** 10.1186/s13045-023-01488-4

**Published:** 2023-08-03

**Authors:** Xinyuan Liu, Juanjuan Zhao, Xiangqian Guo, Yongping Song

**Affiliations:** 1 Institute of Biomedical Informatics, Bioinformatics Center, Henan Provincial Engineering Center for Tumor Molecular Medicine, School of Basic Medical Sciences, Henan University, Kaifeng, 475004 Henan China; 2 Department of Hematology, The First Affiliated Hospital of Zhengzhou University, Zhengzhou, 450052 Henan China

**Keywords:** Non-Hodgkin's lymphoma, Bispecific antibody, CD20, CD3, EPCORE NHL-1

## Abstract

Multiple bispecific antibodies (bsAbs) have been approved for cancer immunotherapy. Several CD20 × CD3 bsAbs have demonstrated significant anti-B-cell non-Hodgkin lymphoma (NHL) activity by engaging T cells to target CD20^+^ NHL cells in clinical trials. Mosunetuzumab, epcoritamab and glofitamab have been approved recently for B-cell NHL therapy. In this study, we summarized several latest reports on CD20 × CD3 bsAbs for the therapy of B-cell NHL from the ASCO 2023 annual meeting (ASCO2023).

To the editor

Several CD20 × CD3 bispecific antibodies (bsAbs) have demonstrated significant anti-B-cell non-Hodgkin lymphoma (NHL) activity by engaging T cells to target CD20^+^ NHL cells in clinical trials. Mosunetuzumab (Mosun), epcoritamab (Epcor) and glofitamab (Glofit) have been approved recently for refractory/relapsed (R/R) B-cell NHL therapy. We summarized several latest reports on CD20 × CD3 bsAbs from the ASCO 2023 annual meeting.

## Properties of CD20 × CD3 bsAbs

Glofit is a full-length IgG1 bsAb with a 2:1 molecular configuration of anti-CD20 and anti-CD3 (Table 1). The Fc of Glofit has a PG LALA mutation, resulting in the loss of the Fc-FcγRs interaction while retaining its binding ability to FcRn [1]. This particular structure possesses a longer half-life of 10 days when compared to earlier generation of bsAbs. Glofit is currently being investigated as a single agent and in combination regimens [2–4].

**Table 1:** Properties of CD20 × CD3 bispecific antibodies

Agent	Glofitamab	Mosunetuzumab	Epcoritamab
Length	Full-length IgG1	Fully humanized Full-length IgG1	Full-length IgG1
Configuration	Anti-CD20: anti-CD3 = 2:1	Anti-CD20: anti-CD3 = 1:1	Anti-CD20: anti-CD3 = 1:1
Fc mutation	PG LALA	N297	L234F, L235E, and D265A
Administration	IV	SC/IV	SC
Dosing	Step-wise	Step-wise	Step-wise
Depleting of B cells prior to bsAb	Yes	No	No
Combined with CD20 mAbs	Yes	No	Yes
References	[1, 4]	[5, 6]	[5, 9]

bsAb, bispecific antibody; Epcor, epcoritamab; Glofit, glofitamab; IV, intravenous; Mosun, Mosunetuzumab; SC, subcutaneous

Mosun is a fully humanized IgG1 bsAb (Table 1). Mosun was administered as a fixed-duration regimen with step-up dosing to minimize cytokine release syndrome (CRS). The intravenous (IV) formulation has been approved, while subcutaneous (SC) Mosun is still in trials [5]. Mosun has been evaluated in clinical trials as a monotherapy or in combination regimens for the treatment of B-cell NHL [6–8].

Epcor is another IgG1 bsAb. It was designed to bind to a unique epitope on CD20 antigen, allowing co-binding of other anti-CD20 agents (Table 1) [5]. Compared with IV administration, SC Epcor (also known as GEN3013) demonstrated comparable bioavailability and B-cell depletion in cynomolgus monkeys [9].

## Efficacies of CD20 × CD3 bsAbs as a single agent

In a phase II clinical trial for patients (pts) with R/R large B cell lymphoma (LBCL), obinutuzumab was administered prior to Glofit monotherapy to first reduce the tumor load [2, 4]. Glofit was given at a step-up dosing regimen. In the latest update, the complete response (CR) and overall response rates (ORR) were 38% and 59%, respectively, with a median follow-up (mFU) of 20.1 months (m) (Table 2). Notably, prior CAR T cell therapy did not adversely affect ORR. The median duration of CR was 24.1 m [95% CI: 19.8–not reached (NR)]. Among pts who received doses lower than recommended but ≥ 10 mg, the CR duration was extended to 30.1 m. The 18-m overall survival (OS) rate was 41%. CRS was the most frequently reported adverse event (AE). Glofit was recently approved for R/R LBCL.

**Table 2:** 2023 ASCO updates from clinical trials of CD20 × CD3 bispecific antibodies for lymphoma therapy

Regimen	Epcor	Glofit	Epcor + R-CHOP	Glofit + Pola-R-CHP	Epcor + R^2^
Lymphoma	R/R LBCL	R/R LBCL	1L high-risk DLBCL	1L DLBCL	R/R FL
Pts	157	154	47	24	109
mFU	20 m	20.1 m	11.5 m	5.1 m	8.8 m
	(range: 0.3+–28.2)	(range: 0–32)	(range: 0.8–15.5)	(range: 3–8)	(range: 1.2–18.5)
ORR	63.1%	59%	100%	100%	97%
CR/CMR	CR: 39.5%	CR: 38%	CMR: 76%	CMR: 76.5%	CMR: 86%
mDoCR	20.8 m	24.1 m (95% CI 19.8–NR)	NR	NR	NR
PFS	87.2% at 1 year in pts with a CR	80% at 1 year in pts with a CR at EOT	NR	NR	NR
OS	mOS:18.5 m (95% CI 11.7–NR)	41% (18-m) (95% CI 32.1–49.3)	NR	NR	NR
CRS	51%	64%	60%	13%	48%
G1–2	48%	60%	57%	13%	46%
G ≥ 3	3%	4%	2%	0	2%
NAE/ICANS	ICANS: 6%	NA	ICANS: 2%	NAE: 46%	ICANS: 2%
G1–2	6%		2%	42%	2%
G ≥ 3	1%		0	4%	0%
Clinical trial number	NCT03625037	NCT03075696	NCT04663347	NCT03467373	NCT04663347
References	[10]	[4]	[11]	[3]	[12]

CMR, complete metabolic response; CR, complete response; CRS, cytokine release syndrome; DLBCL, diffuse large B-cell lymphoma; EOT: end of treatment; Epcor, epcoritamab; FL, follicular lymphoma; G: grade; Glofit, glofitamab; ICANS, immune effector cell‐associated neurotoxicity syndrome; m, months; mDoCR, median duration of CR; mFU, median follow-up; mOS: median OS; Mosun, Mosunetuzumab; NA: not available; NAE, neurological adverse events; NR, not reached; ORR, overall response rate; OS, overall survival; PFS: progression-free survival; Pola, polatuzumab vedotin; Pts, patients; R-CHOP, rituximab, cyclophosphamide, doxorubicin, vincristine, and prednisone; R/R, relapsed/refractory; 1L, 1 line

In the EPCORE NHL-1 trial for R/R LBCL, single-agent Epcor was administered with a step-wise dosing regimen [10]. The CR and ORR were 39.5% and 63.1%, respectively (Table 2). Median OS was 18.5 m. CRS occurred in 51% of pts, and immune effector cell‐associated neurotoxicity syndrome (ICANS) occurred in 6% of pts. Epcor was recently approved for R/R LBCL.

## Efficacies of CD20 × CD3 bsAbs in combination regimens

In the EPCORE NHL-2 trial, Epcor was combined with R-CHOP in adults with untreated high-risk DLBCL [11]. In the latest report, 47 pts had received Epcor 48 mg + R-CHOP with a mFU of 11.5 m (Table 2). All pts achieved a response (100%), and 76% achieved a complete metabolic response (CMR). The CMR occurred in 82% double/triple-hit pts. CRS occurred in 60% of pts, and 1 pt experienced ICANS.

In the EPCORE NHL-2 trial, SC Epcor was combined with R + lenalidomide in treating R/R follicular lymphoma (FL) [12]. One hundred and nine pts were treated with a mFU of 8.8 m. The CMR and ORR were 86% and 97%, respectively (*n* = 101) (Table 2). CRS occurred in 48% of pts, while 2 pts developed ICANS.

Glofit was employed in combination with polatuzumab vedotin (Pola) plus R-CHP for untreated DLBCL pts, with Pola-R-CHP given on Day 1 of each cycle (C) and Glofit administered in C2–C6 at a step-up dosing [3]. The CMR and ORR occurred in 76.5% and 100% of the 24 enrolled pts after a mFU of 5.1 m (Table 2). CRS and neurological AEs occurred in 13% and 46% pts, respectively.

In addition to the above trials, several clinical trials on CD20 × CD3 bsAbs in combination regimens are enrolling now. These include SC Mosun + IV Pola in pts with R/R NHL [8], SC Mosun with lenalidomide augmentation in pts with untreated FL and marginal zone lymphoma [7], and SC Epcor + R-CHOP in pts with newly diagnosed DLBCL [13].

In summary, Mosun, Epcor and Glofit have been approved recently for R/R B-cell NHL therapy. SC administration of Epcor offers convenience. CD20 × CD3 bsAbs in combination regimens are in clinical trials.

## Data Availability

The material supporting the conclusion of this study has been included within the article.

## Abbreviations

AE: Adverse events
ASCO: American Society of Clinical Oncology
bsAb: Bispecific antibody
CMR: Complete metabolic response
CR: Complete response
CRS: Cytokine release syndrome
DLBCL: Diffuse large B-cell lymphoma
Epcor: Epcoritamab
FL: Follicular lymphoma
Glofit: Glofitamab
ICANS: Immune effector cell‐associated neurotoxicity syndrome
IV: Intravenous
m: Months
mDoCR: Median duration of CR
mFU: Median follow-up
Mosun: Mosunetuzumab
NHL: Non-Hodgkin lymphoma
NR: Not reached
ORR: Overall response rate
OS: Overall survival
Pola: Polatuzumab vedotin
pts: Patients
R-CHOP: Rituximab, cyclophosphamide, doxorubicin, vincristine, and prednisone
R/R: Relapsed/refractory
SC: Subcutaneous
1L: 1 Line
